# Reproducible segmentation of white matter hyperintensities using a new statistical definition

**DOI:** 10.1007/s10334-016-0599-3

**Published:** 2016-12-09

**Authors:** Soheil Damangir, Eric Westman, Andrew Simmons, Hugo Vrenken, Lars-Olof Wahlund, Gabriela Spulber

**Affiliations:** 1 Department of Neurobiology, Care Sciences and Society, Karolinska Institutet, Hälsovägen 7, Huddinge, 14157 Stockholm, Sweden; 2 Institute of Psychiatry, King’s College London, Box P089, De Crespigny Park, London, SE5 8AF UK; 3 Department of Physics and Medical Technology, VU University Medical Center, De Boelelaan 1118, 1081HZ Amsterdam, The Netherlands; 4 Department of Radiology and Nuclear Medicine, VU University Medical Center, De Boelelaan 1118, 1081HZ Amsterdam, The Netherlands

**Keywords:** Segmentation, White matter hyperintensities, White matter lesion, Multimodal segmentation

## Abstract

**Objectives:**

We present a method based on a proposed statistical definition of white matter hyperintensities (WMH), which can work with any combination of conventional magnetic resonance (MR) sequences without depending on manually delineated samples.

**Materials and methods:**

T1-weighted, T2-weighted, FLAIR, and PD sequences acquired at 1.5 Tesla from 119 subjects from the Kings Health Partners-Dementia Case Register (healthy controls, mild cognitive impairment, Alzheimer’s disease) were used. The segmentation was performed using a proposed definition for WMH based on the one-tailed Kolmogorov–Smirnov test.

**Results:**

The presented method was verified, given all possible combinations of input sequences, against manual segmentations and a high similarity (Dice 0.85–0.91) was observed. Comparing segmentations with different input sequences to one another also yielded a high similarity (Dice 0.83–0.94) that exceeded intra-rater similarity (Dice 0.75–0.91). We compared the results with those of other available methods and showed that the segmentation based on the proposed definition has better accuracy and reproducibility in the test dataset used.

**Conclusion:**

Overall, the presented definition is shown to produce accurate results with higher reproducibility than manual delineation. This approach can be an alternative to other manual or automatic methods not only because of its accuracy, but also due to its good reproducibility.

**Electronic supplementary material:**

The online version of this article (doi:10.1007/s10334-016-0599-3) contains supplementary material, which is available to authorized users.

## Introduction

White matter hyperintensities (WMH) are radiological findings on MR images that are classically defined as areas with relatively high signal intensities on T2-weighted images (T2) and low intensities on T1-weighted images (T1). The presence and spatial patterns of WMH on MRI and the appearance of these changes are important for studying pathology and for prospective clinical practice including diagnosis, following progression, and monitoring treatments.

Table 1 presents the desirable characteristics of an algorithm for the automatic detection of WMH to be widely usable. Although many automatic methods have been proposed in the last 20 years [1–10], no single method is widely employed, nor does it satisfy all desirable characteristics of being widely used [11]. An important source of the imperfect performance of automated WMH segmentation methods is the attempt to solve a problem for which there is no unique solution. In other words, although WMH are visually appreciable, expert human raters do not agree either on the general definition of WMH or on the precise segmentation of individual scans, resulting in automatic WMH segmentation methods that are aimed at a moving target. The problem of segmenting WMH as viewed in this way is an *ill*-*posed* problem [12]. The *ill*-*posed* characteristic sets WMH segmentation apart from many other segmentation problems, in which much closer agreement between experts is reached; this is why there are widely used and accepted methods for those other segmentation problems. We believe that if the WMH segmentation problem were *well*-*posed*, it would served as a foundation for a stable computer solution. Although the previous approaches can be and have been useful in numerous scenarios, a new approach is needed in order to achieve a general solution.

**Table 1:** Desirable features for a WMH segmentation algorithm and their availability in different methods

	Zijdenbos et al. [4]	Shiee et al. [5]	Raniga et al. [6]	Damangir et al. [7]	Schmidt et al. [8]	Steen-wijk et al. [9]	Guizard et al. [10]
Technique used	ANN	Clustering	OD	SVM	OD and RG	kNN	RI
No manual editing	No	Yes	Yes	No	Yes	No	No
Any conventional MRI sequences	No	No	?	?	No	No	Yes
Independent of scanning parameters	?	Yes	Yes	?	Yes	Yes	?
Handle diffuse dirty white matter	No	No	No	No	No	No	No
Handle partial volumes	No	No	No	No	No	No	No
Multi-center datasets	?	No	No	No	No	No	?
Duration	–	2 h	–	45 m	1.5 h	3 h	1 h
Publicly available	No	Yes	No	Yes	Yes	No	No

*ANN* artificial neural network, *OD* outlier detection, *SVM* support vector machines, *RG* region growing, *kNN* k-nearest neighbors algorithm, *RI* rotation invariant features, *Yes* satisfied (proved), *?* Argued in discussion, not proved, *No* does not satisfy, – does not mention

In the present work, the problem of WMH segmentation has been reformulated as a *well*-*posed* problem. An easy-to-implement statistical test has been proposed to compare the local image intensity to the global intensity as a reformulation of the current descriptive definition of WMH. The concrete statistical definition for WMH, which enables segmentation independent of manual reference and scanning parameters, has been shown to yield results with the same quality as the traditional supervised machine learning method.

In this study, we show that the proposed *well*-*posed* reformulation addresses the same question as the traditional approach: the proposed definition has been shown to be aligned with the traditional visual description by comparing the results using all combinations of input sequences (e.g., Fluid-attenuated inversion recovery (FLAIR), T1, T2 and T1) with manual delineation. These segmentations have then been compared with Lesion TOADS and LST [5, 8], two other available state-of-the-art methods, which work with T1 and FLAIR, to assess whether or not the proposed statistical definition can be used in place of automatic methods that aim to replicate traditional visual descriptions of WMH. Segmentation using different combinations of input sequences are cross-compared to one another (e.g., segmentation using FLAIR and T1 compared with the one using T2 and T1) to simulate a scenario in which different imaging data protocols were used in a multi-center study.

The experiment described in this paper uses a dataset with four widely used MRI sequences (T1, T2, FLAIR, and PD) and manual WMH delineation. After describing the dataset, the proposed definition of WMH is presented followed by step-by-step descriptions of all necessary pre-processing and its implementation. Then, the experimental setup and its results are presented before discussing the method and implication of the results.

## Materials and methods

### Subjects

Data used in the preparation of this paper were obtained from the Kings Health Partners-Dementia Case Register (KHP-DCR) in the UK. MRI scans of 119 subjects (Alzheimer’s disease (AD), mild cognitive impairment (MCI), and healthy controls) were used from the KHP-DCR. The AD diagnosis was made according to the Diagnostic and Statistical Manual for Mental Diagnosis (fourth edition) and MCI was defined according to the Petersen criteria [13]. Subjects were 76.4 ± 7.4 years old, 56% female, and had 12.0 ± 4.3 years of education and a mini-mental state examination (MMSE) scores of 26.5 ± 4.8.

The imaging protocol included the following sequences: sagittal 3D T1-weighted MPRAGE, axial proton density (PD), T2-weighted fast spin echo image, and 2-D FLAIR. All images had been acquired with a 1.5 Tesla scanner and had full brain and skull coverage. Quality control was performed according to the AddNeuroMed procedure [14]. Table 2 shows the detailed sequence information.

**Table 2:** Description of imaging pulse sequence protocols

	Slice thickness (mm)	Slice gap (mm)	Matrix	Field of view	Echo time (ms)	Repetition time (ms)	Inversion time (ms)	Flip angle (deg)
MPRAGE	1.2	1.2	192 × 192	240	3.80	8.6	1000	8
PD	3	3	256 × 256	240	10.58	3000	0	90
T2	4	5.5	512 × 512	240	88.16	5000	0	90
FLAIR	4	5.5	320 × 320	240	160.70	10,000	2500	90

In the rest of the paper, T1 refers to the T1-weighted MPRAGE and T2 refers to the T2-weighted image.

### White matter hyperintensities definition

The common definition of WMH is based on their visual properties on specific pulse sequences (hyper- or hypo-intensities), which has been proved to be insufficiently reproducible for large multi-center studies [11].

We incorporated the common definition of WMH in a new statistical definition that can be robustly measured. This study defines WMH as areas where their local image histograms are significantly different from the expected normal local histogram on the one-tailed test.

This proposed statistical definition differs from machine learning methods and outlier detection methods, in which statistical features of manually delineated WMH are captured in a supervised or unsupervised way. In contrast, the proposed method defines WMH independent of manual delineation, and it is only based on the common definition.

A one-tailed Kolmogorov–Smirnov test has been used as the statistical test as shown in Eq. 1:

1 $$\begin{array}{*{20}c} {D^{ + } = \sup \left( {F_{1} \left( i \right) {-} F_{2} \left( i \right)} \right)} \\ {D^{ - } = \sup \left( {F_{2} \left( i \right) {-} F_{1} \left( i \right)} \right)} \\ \end{array}.$$

where, *F*_1_ is the cumulative local histogram and *F*_2_ is the expected normal local histogram at index *i*.

The test statistic distribution is empirically calculated using permutation of all test statistics for voxels in an evidently normal brain. In the present study, significance level 0.05 was used, and the expected local histograms of normal brain were calculated for each voxel as the average of the local histograms of evidently normal voxels in the same subject. Figure S1 in the supplementary material illustrates sample local histograms for different brain tissue types and image sequences. Evidently, normal voxels are calculated in two steps as described in the section “Calculating evident normal brain”.

### Image processing

#### Preprocessing

The aim of the preprocessing is to register all input pulse sequences together, correct them for inhomogeneity, and estimate initial brain segmentation as white matter (WM), gray matter (GM) and cerebrospinal fluid (CSF). Preprocessing comprises the following steps performed with the FSL package (http://fsl.fmrib.ox.ac.uk/fsl):

1. Intra-subject registration using rigid 3-D transformation with mutual information (FSL FLIRT [15] http://fsl.fmrib.ox.ac.uk/fsl/fslwiki/flirt).
2. Skull stripping (FSL BET [16] http://fsl.fmrib.ox.ac.uk/fsl/fslwiki/bet).
3. Inhomogeneity correction for all registered input images using the N3 algorithm [17].
4. Brain tissue segmentation (FSL FAST [18] http://fsl.fmrib.ox.ac.uk/fsl/fslwiki/fast) into GM, WM, and CSF voxels.
5. Refining brain tissue segmentation: GM voxels that are bright on either FLAIR or T2 images (top 15% voxels of GM intensity histogram) are labeled as suspicious voxels. Suspicious voxels surrounded by mostly WM are labeled as WM and voxels surrounded by mostly GM are labeled as GM.

**Fig. 1:**
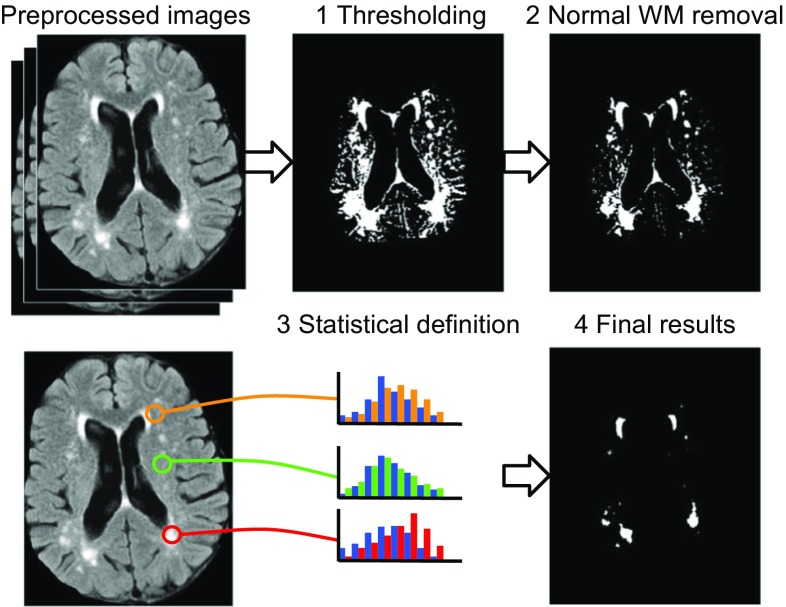
Results after each step of CASCADE. *Step 1* results after thresholding, *Step 2* results after second thresholding and morphological filter, *Step 3* testing all voxels in the results of Step 2 against the statistical definition of WMH to generate the WMH confidence map, *Step 4* thresholding WMH confidence map at the desired level to produce a binary WMH mask

The results of the preprocessing step were then used as the input to the rest of the procedure (as input sequences in Fig. 1).

#### Calculating evident normal brain

The proposed definition depends on the perception of a normal brain. In this paper, evidently normal voxels are simply calculated in two steps:

1. Heuristic thresholding is used to capture the bottom percentile (hypo-intense area) of voxels for FLAIR (45%), T2 (50%), and PD (65%) and the upper percentile (hyper-intense area) of voxels for T1 (15%). The thresholding was performed independently on each sequence and applied three times after smoothing the image with Gaussian kernels of size 1, 2, and 3 mm. The final threshold mask is the intersection of all masks for each sequence and scale (i.e., AND operation). This step should generally be expected to remove some of non-WMH voxels while keeping all WMH voxels (results in Step 1 in Fig. 1).
2. The masks generated in the first step are used as the training for a support vector machine algorithm (SVM) with a Gaussian kernel, and then the SVM is reduced to a single-node SVM using the reduction algorithm described by Schölkopf et al. [19] The voxels that are classified by the single-node SVM is the output mask for this step.

Voxels captured by all the masks above (i.e., all masks AND-ed together) are considered as an evidently normal brain mask. A Proper Closing morphological filter is then used to include small holes and missing voxels in the evidently normal brain before using the mask in the WMH definition. Proper Closing is defined as in Eq. 2, using initial mask (M), morphological closing (C), and opening (O) functions with a 2-mm spherical structuring element (results in Step 2 in Fig. 1).

2 $${\text{Proper}} \;{\text{closing}}\left( M \right)\;\underline{\underline{\text{def}}} \;M \wedge O\left( {C\left( {O\left( M \right)} \right)} \right)$$

## Validation procedure

Manual delineation of WMH was used as a reference to evaluate the segmentation results. Manual delineation was performed by a trained radiologist according to the protocol described in [20]. WMH were delineated on the FLAIR images and then registered to other sequences using the same transformation calculated in the preprocessing steps. Having the manual delineation as a reference, the validity of the proposed approach was investigated in three experimental settings:

First, the segmentation accuracy was assessed by comparing the results directly to manual delineation. The segmentations using all 15 possible different sequence combinations of T1, T2, FLAIR, and PD have been considered for comparison.

Second, the segmentations were compared to that of Lesion TOADS and LST [5, 8], two other publicly available software methods on our dataset.

Third, in order to investigate the robustness and generalizability of the proposed statistical definition, the segmentations produced using different combinations of input sequences were compared to one another. This comparison helps to predict the expected similarity should the method be used in a multi-center study with different image modality combinations (e.g., one center with T1 and FLAIR images, and another center with T1 and T2 images).

In all these three situations, fixed significance levels of 0.05 were used, and three measures were calculated to compare two segmentations:

1. Correlation coefficient: to measure similarity of total estimated volume.
2. False negative rate (FNR) and false discovery rate: to measure the types of errors in segmentation (i.e., missing or over estimating WMH)
3. Dice coefficient: to compare the extent to which two segmentations overlap. The Dice coefficient [21] is defined in Eq. 3 as twice the total volume of WMH that was labeled by both methods, divided by the sum of the total volumes obtained by them, where *S*_1_ and *S*_2_ are the two segmentations to compare:

3 $${\text{Dice}}\;\underline{\underline{\text{def}}} \;\frac{{2 \times \left( {S_{1} \mathop \cap \nolimits S_{2} } \right)}}{{S_{1} + S_{2} }}.$$

Statistical analysis and plotting were performed using MATLAB R2014B. The Pearson correlation coefficient was used for calculating correlation between volumes.

## Results

### Comparison to manual delineation

Table 3 describes the distribution of the WMH load in the dataset. The WMH size varies between subjects in this dataset, capturing different levels of involvement from very small patches of WMH to a very high load of WMH, making this a useful dataset for the current development project.

**Table 3:** Descriptive statistics of estimated volume of WMH using different input sequences and their false negative (FNR) and false discovery rate (FDR)

	Volume (cc)					FNR (%)	FDR (%)
	Minimum	25%	Median	75%	Maximum		
Manual (on FLAIR)	0.447	6.805	20.506	33.006	150.290	–	–
PD	0.256	3.421	10.510	17.213	81.685	58.1	19.9
T1	0.275	4.059	12.234	22.093	99.503	47.5	17.2
PD + T1	0.313	4.343	13.087	20.901	95.043	44.8	15.6
T2	0.442	6.807	20.219	32.512	149.071	15.1	14.8
FLAIR	0.454	6.828	20.181	33.420	150.048	13.3	13.4
T1 + FLAIR	0.442	6.791	20.308	33.391	147.853	7.9	8.1
T1 + T2	0.445	6.737	20.703	33.277	148.391	12.3	12.2
PD + FLAIR	0.443	6.787	20.334	32.970	149.633	12.5	12.4
PD + T2	0.444	6.887	20.516	33.029	152.026	15.2	15.5
T2 + FLAIR	0.451	6.699	20.654	33.018	149.734	14.5	14.4
T1 + FLAIR + PD	0.454	6.798	20.871	33.272	148.040	8.1	8.0
T2 + FLAIR + PD	0.442	6.658	20.432	33.118	149.647	16.5	15.6
T1 + T2 + PD	0.449	6.735	20.266	32.943	152.826	12.1	11.9
T1 + T2 + FLAIR	0.455	6.712	20.456	33.039	151.451	8.4	8.2
T1 + T2 + PD + FLAIR	0.441	6.811	20.352	33.369	147.593	8.7	8.5

Since the range of WMH load is large, for all the figures of the “Results” section, we report the ratio between measured WMH volumes and the volumes of the manual segmentations to obtain values in the same range for all subjects to facilitate visual comparison. In all box-and-whisker plots, the highlighted band specifies the estimated manual performance reported in the literature [4, 22, 23] (i.e., manual inter-rater agreement).

**Fig. 2:**
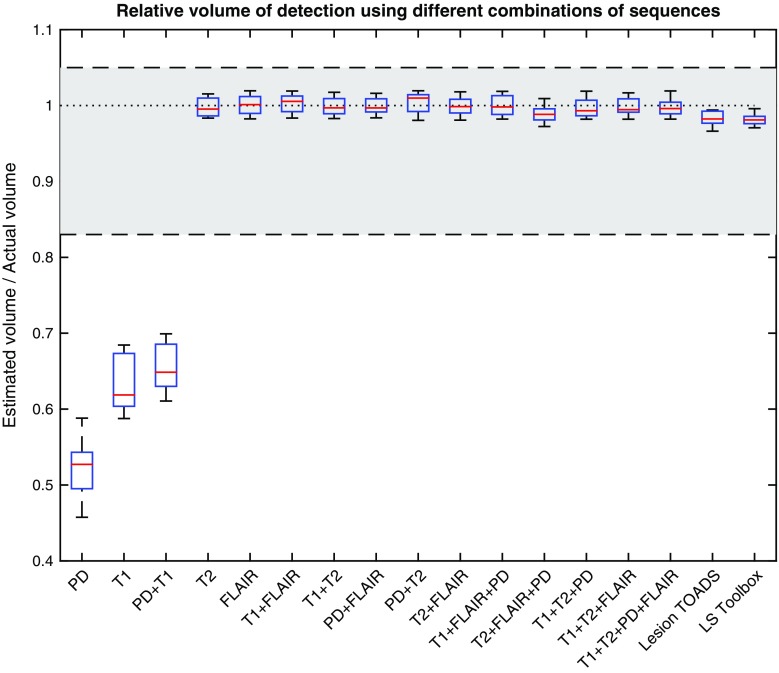
Ratios between estimated WMH volume and manual delineated WMH volume; estimated volume calculated using Lesion TOADS, LS Toolbox and CASCADE with different combinations of input sequences. *Highlighted area* refers to the expected range of human performance based on reported inter-rater agreement

Figure 2 compares the volumes obtained from different combinations of input sequences to those obtained by manual delineation. It shows that all combinations of input sequences produce WMH volumes that are sufficiently close to the manual delineation, except for PD, T1, and PD + T1.

**Fig. 3:**
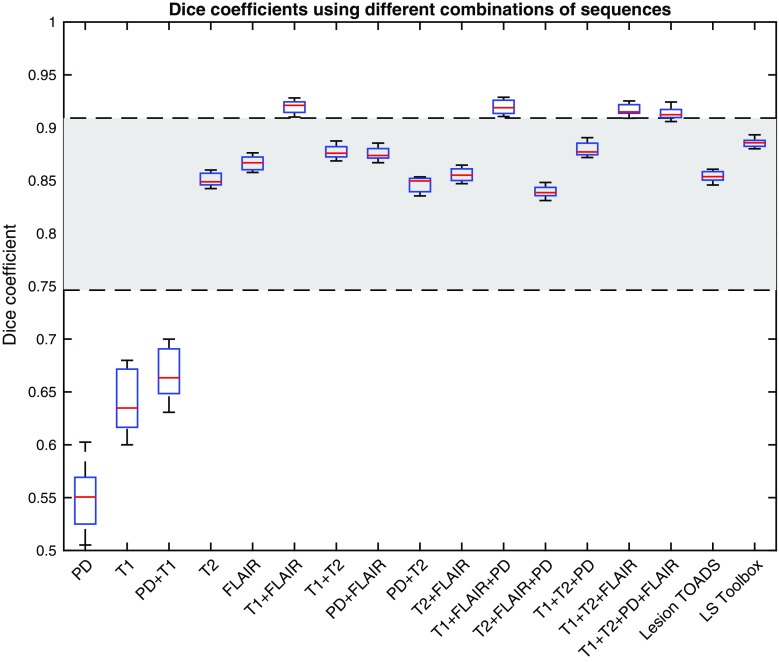
Dice coefficients comparing estimated WMH masks from Lesion TOADS, LS Toolbox, and CASCADE using different combinations of input sequences with a manually delineated WMH mask. *Highlighted area* refers to the expected range of human performance based on reported inter-rater agreement

Figure 3 shows the Dice coefficient between the results of the proposed method and manual delineation. Similar to the results of volume correlation, all combinations of sequences perform comparable to manual delineation.

**Fig. 4:**
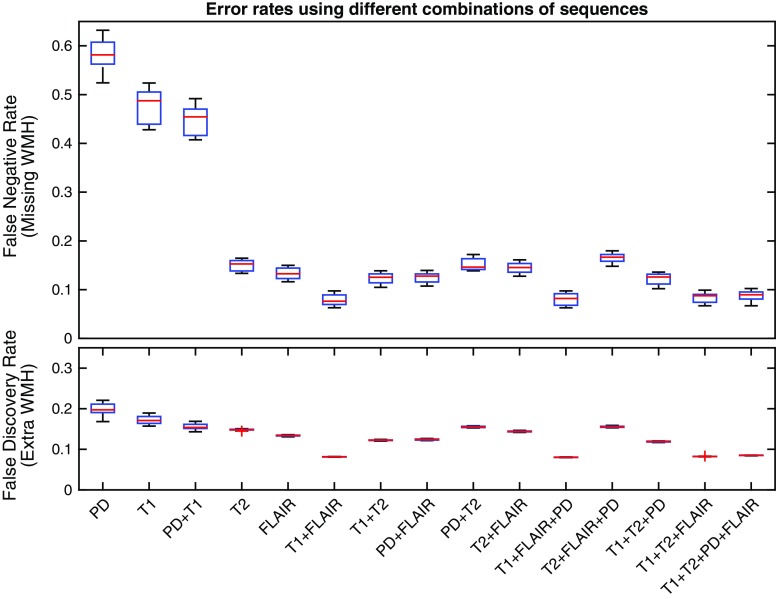
Error rate illustrated by false positive rate (FPR) and false discovery rate (FDR); calculated using different combination of input sequences

Figure 4 shows the error rates using different combination of sequences. In our experiment, false negative and false positive rates were in the same range for all combinations that have T2 or FLAIR. Even though WMH volumes from all input sequences strongly correlates with the volume from manual delineation, results using only T1 or PD suffer from a large false negative rate (i.e., missing WMH detection).

**Fig. 5:**
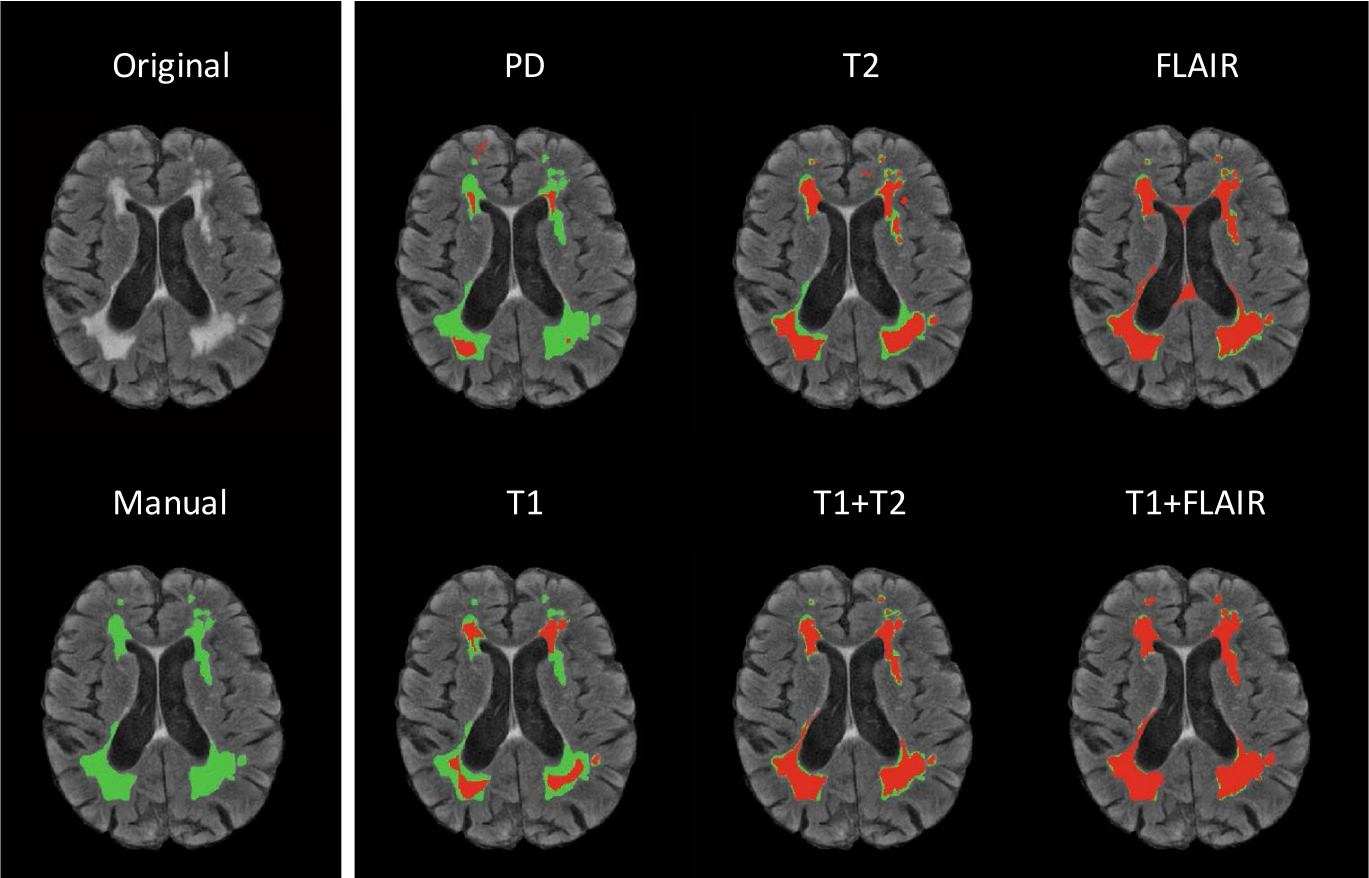
A sample slice overlaid with CASCADE output given different input sequences as input. *Blue* Manual delineation. *Red* CASCADE output

Finally, Fig. 5 illustrates a sample segmentation using the proposed method for visual reference.

### Comparison to other methods

The results of the segmentation using different combinations of sequences were also compared to two available state-of-the-art methods: Lesion TOADS and LST [5, 8]. T1 and FLAIR were used as input pulse sequences to both methods. As Fig. 2 shows, both Lesion TOADS and LST produce volumes very close to volumes from manual delineation with a slight underestimation. Figure 3 shows the Dice coefficient between these methods and manual delineation and confirms both methods can produce the results in an acceptable range. Although these methods produced acceptable results, in our experiment for older populations, our method could surpass their Dice similarity using most of the combinations of input sequences.

### Multicenter analysis simulation

A performance bottleneck for analyzing multicenter data is that each center may have its own imaging routine, unless optimized using a multi-center protocol.

In order for a method to be usable in studies with a different imaging protocol (e.g., some subjects with T1 + FLAIR and others with T1 + T2 sequence) the segmentation with different input sequence should be comparable. Since high similarity between manual segmentation and the first (e.g. T1 + FLAIR) and second (e.g. T1 + T2) sequence combination does not guarantee high similarity between first and second segmentation (see supplementary Fig. S2), in this experiment, the output segmentation from different combinations of input sequences were compared against one another, e.g., the results obtained using T1 + FLAIR were compared with those using T1 + T2. In this experiment, the results from manual delineation are not taken into account and the similarity of the segmentation in different scenarios has been assessed.

**Fig. 6:**
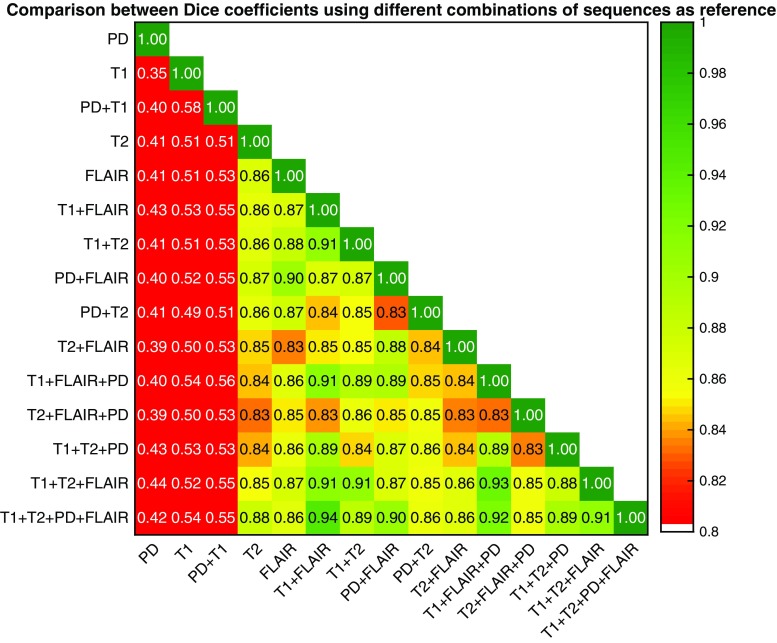
Dice coefficients comparing WMH masks when measured using different input sequences and comparing results from CASCADE using different input sequences to one another

Figure 6 shows the Dice coefficient when comparing the results from different input sequences. The value in each cell corresponds to the expected performance measure when comparing results from two hypothetical centers. It can be observed that in the presence of the T2 or FLAIR sequences, the results from two different centers can be comparable and the expected Dice coefficient is always more than 0.8. In particular, a comparison of the T1 + FLAIR and T1 + T2 combinations, a common scenario in multicenter studies, results in a Dice coefficient of 0.91.

For volume comparison, Fig. S3 (in the supplementary material) illustrates the ratio between the WMH volumes obtained from different sequence combinations.

## Discussion

White matter hyperintensities are referred to as areas with unusually high signal intensities on FLAIR or T2-weighted MRI and/or unusually low attenuation on T1-weighted images. This description has been used for WMH segmentation, using either manual delineation or automatic segmentation methods that aimed to replicate manual delineation. Manual guidelines are subject to interpretation and so are automatic methods based on machine learning that explicitly or implicitly rely on manual delineation. In order to avoid the use of any explicit or implicit interpretation, we attempted to provide a concrete statistical definition of the WMH that implies no human decision. This definition will also improve future software development as it directly addresses some important issues:

- Usable with any available pulse sequence combination without any modification since it works with one pulse sequence at a time.
- Produces comparable results with different input pulse sequences, and thus can be used in multi-center studies.
- Although the issue of partial volumes and ambiguous WMH borders are still not completely solved, because of the probabilistic nature of our definition, the volume and the shape of the WMH can be estimated from the probabilistic maps that are output.

Validation analyses have been performed using different sequence combinations as input in order to assess segmentation performance. We found that any combination of pulse sequences containing either FLAIR or T2 images produces valid and reliable results, especially if the main interest is to obtain regional volumes, shapes, or distribution of the WMH. However, using both T2 and FLAIR segmentation does not cause a dramatic improvement in quality. Including T1 images in the input sequences significantly increases the accuracy of the results by boosting the accuracy of brain tissue segmentation. PD images should be added only if the other modalities cannot result in a sufficiently good brain extraction.

Using the statistical definition of the WMH, we also investigated the performance of two other well-established methods for WMH segmentation: Lesion TOADS and LST [5, 8]. We have shown that the WMH volume and Dice coefficient of the proposed statistical definition is slightly higher than those methods on our dataset.

In order to facilitate further comparisons, we have also implemented the definition and algorithms described in this paper in an open-source software package called CASCADE, and made it publicly available (appendix in supplementary material and http://ki.se/en/nvs/cascade). Since the main idea of this study is to segment WMH, we keep the implementation simple by using only FSL utilities for pre-processing and finding an evidently normal brain that our definition is based upon. Specifically, in this study, we use a simple method for finding an evidently normal brain based on adaptive thresholding, which leads to reliable results (Figs. 2, 3). We also substituted the results of Lesion TOADS and LST segmentation for detecting an evidently normal brain (Fig. S4 in the supplementary material), and showed that the output was robust independent of the quality of the initial segmentation (Fig. S5 in the supplementary material). This suggests that our statistical definition can be used as an add-on to the current methods (including manual, semi-automatic, and automatic) to ensure reproducibility of the results.

In this study, a fixed significance level of 0.05 was used for all sequence combinations to ensure implicit and explicit independence from manual delineation. One may want to optimize the significance level to maximize the Dice coefficient for different sequences, which in the present dataset would lead to 0.04–0.06 (0.046 ± 0.008). However, we believe applying such an optimization defeats the purpose of the method which is to approach the problem of WMH segmentation as a *well*-*posed* problem without any implicit or explicit dependency on manual delineation. Therefore even though changing the significance level may increase the accuracy for particular datasets, we believe the significance level should be set outside the image processing pipeline.

### Comparing other approaches

One of the main contributions of this study is the presentation of a new approach for the problem of WMH segmentation in which any implicit or explicit connection to manual WMH delineation is circumvented.

So far, three main approaches have been used to measure WMH in the literature: manual [24–28], supervised [4, 7, 9] and unsupervised [5, 6, 8] machine learning methods (Table S1 in the supplementary material). Using the currently accepted definition of WMH, manual delineation has been used for the WMH assessment [24–28]. However, low reproducibility and the need for human interaction have made it not feasible in large multi-center studies. Furthermore, these various guidelines have shown inconsistent correlations with different clinical features [28, 29]. Thus, computerized machine learning approaches emerged as tools for rapid and accurate segmentation of WMH.

Machine learning-based segmentation is performed using either supervised or unsupervised learning techniques. However, both supervised and unsupervised methods rely on manual delineation data either for input or target segmentation. Supervised learning methods explicitly involve human input, and thus, to some degree have the same fundamental problem of manual measurement. Unsupervised methods, based on either clustering or outlier detection methods, are also implicitly relying on human input by targeting replication of human results. Even though the measurements using supervised and unsupervised methods are relatively reproducible with the least inter-rater disagreement, WMH are defined through the experts’ interactions in each setting where the intra-rater disagreement is still expected to be high. In other words, since the scope of machine learning approaches is to mimic expert delineation, they conceptually carry the same disagreement problem as manual measurements.

On the other hand, the proposed statistical definition does not aim to mimic the manual delineation. Rather, it aims to resemble the general description and be reproducible. The generated reproducible measure of WMH is then observed to be close to manual delineation.

In other words, the main difference between machine learning methods and our proposed method is that machine learning methods are optimized to re-create manual segmentation; however, our statistical definition is optimized to have a WMH measure in the most reproducible way, and the defined WMH measure is similar to the manual segmentation as a byproduct. One may dispute that our approach is not necessarily representative of the underlying pathology, and the results are not the same as what is measured using manual WMH delineation. However, given that the only sources of information about underlying pathologies on MR images are the actual intensities, WMH segmentation based on either guidelines or our definition are indirect measurements of the underlying pathology. Thus, both approaches may have the same source of systematic errors. Nevertheless, the high correlation and similarity between two measures ensures that both approaches are measuring the same underlying entity with different levels of accuracy and reproducibility.

The idea of making a mathematical definition has been reported in the literature. Other researchers have proposed approaches to define WMH mathematically [30]. However, their definitions are complex, and they are not known to remain consistent across different centers. Our statistical definition of WMH uses simple statistics and is robust and reproducible, although it might be slightly inconsistent with experts’ manual definitions. This deviation is anticipated as manual delineations themselves have reportedly 10–32 percent intra-rater disagreement, which approximately equals a Dice coefficient range of 0.76–0.90 [4, 22, 23]. Although some deviation is observed, the segmentation produced with our statistical definition has very low disagreement when different combinations of sequences are used. For instance, the results using T1 + FLAIR have just nine percent disagreement (Dice coefficient 0.91) compared to those obtained from T1 + T2. Low degree of disagreement can be invaluable in multi-center studies where results from datasets with different image modalities need to be compared to one another.

## Conclusion

Reformulating the problem of WMH segmentation as a *well*-*posed* one, our new approach can segment WMH with high accuracy and reproducibility using any combination of MRI sequences. This new approach is applicable for multi-center studies where it is crucial to have high output similarity when comparing results from different datasets.

We showed that segmentation based on the proposed approach has slightly better accuracy than other major methods in the literature; however, since the accuracy is measured against the moving target of manual segmentation, accuracy is not the most crucial indicator of a method. We believe reproducibility of the measurement serves a more important role. Thus, until WMH can be directly measured, reproducible methods such as our concerted definition are favorable to manual delineation.

## Electronic supplementary material

Below is the link to the electronic supplementary material.

Supplementary material 1 (PDF 52 kb)

**Fig. S1** Sample cumulative histogram for different sequences illustrating the histogram of differentpatches of white matter hyperintensities (WMH) and normal appearing white matter in comparison withthe histogram of the evident normal brain (PDF 141 kb)

**Fig. S2** Similarity of two segmentations to a third reference segmentation does not guarantee thesimilarity between the first and the second segmentation. Segmentation 1 and 2 have equal Dicesimilarity index with reference segmentation, but they have A) low similarity; B) average similarity C)high similarity D) very high similarity (PDF 46 kb)

**Fig. S3** Ratio between white matter hyperintensities (WMH) volume when measured using differentinput sequences, comparing results from CASCADE using different input sequences to one another(Volume estimated from input combination indicated in the horizontal axis divided by the one in thevertical axis) (PDF 75 kb)

**Fig. S4** Processing design to analyze the effect of using different algorithms for detecting “evidentlynormal brain” All inputs use T1 and FLAIR as the input sequence. The dashed line is the procedureused in the CASCADE implementation, used to create the results in the paper. AT: Adaptive threshold,SD: Statistical definition (PDF 14 kb)

**Fig. S5** Comparison between the results of WMH segmentation using different methods for detectingevidently normal brain. All inputs use T1 and FLAIR as the input sequence. AT: Adaptive threshold,SD: Statistical definition (PDF 22 kb)

Table S1 (PDF 56 kb)
