# Supplementary material for: Reproducible segmentation of white matter hyperintensities using a new statistical definition
Source: MAGMA. 2016 Dec 9;30(3):227–37. doi: 10.1007/s10334-016-0599-3 (PMC5440501; doi:10.1007/s10334-016-0599-3)
Supplement: Supplementary file 1 — Supplementary material 1 (PDF 52 kb) [file 10334_2016_599_MOESM1_ESM.pdf]

APPENDIX: USING CASCADE STATISTICAL DEFINITION AS A SOFTWARE

The recommended practice for using this method is to use FLAIR if it is available. In the absence of FLAIR, T2 should be used instead. T1 image should always be added if it is available. PD should be added only if the quality of the images is good and other modalities cannot result in a sufficiently good brain extraction. In the case of PD, the contribution of having another source of information can be outweighed by the noise introduced due to low image quality. The CASCADE software package is publicly available at http://ki.se/en/nvs/cascade for installation and through neuGRID4you (European e-infrastructure for neuroimaging) at https://neugrid4you.eu for public use. The current version of the software can use any combination of T1, T2, FLAIR, and PD images and create the WMH segmentation map and brain tissue volumes
