# Supplementary figures and images for: Reproducible segmentation of white matter hyperintensities using a new statistical definition
Source: MAGMA. 2016 Dec 9;30(3):227–37. doi: 10.1007/s10334-016-0599-3 (PMC5440501; doi:10.1007/s10334-016-0599-3)

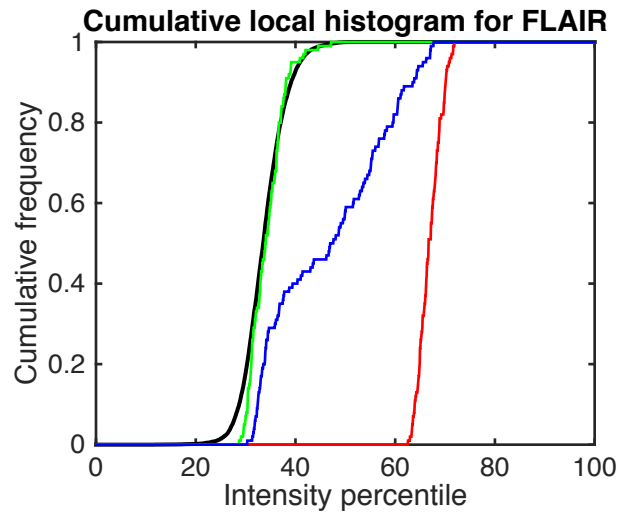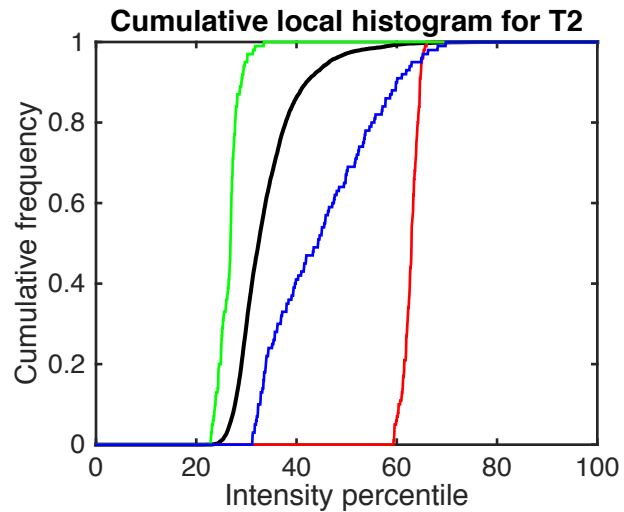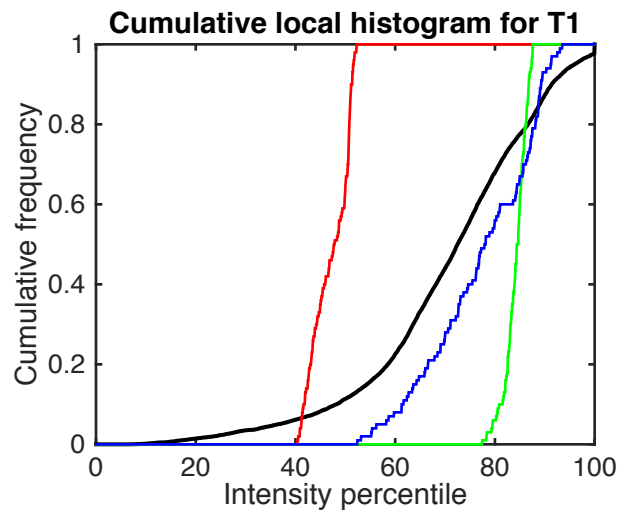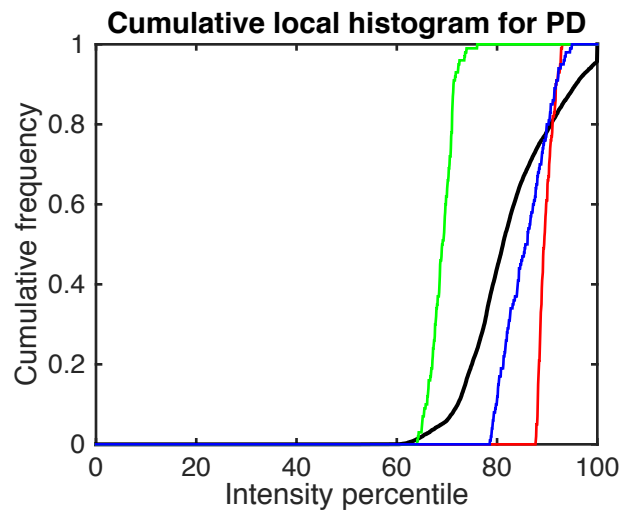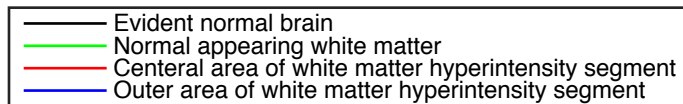

Supplement: Supplementary file 2 — Fig. S1 Sample cumulative histogram for different sequences illustrating the histogram of differentpatches of white matter hyperintensities (WMH) and normal appearing white matter in comparison withthe histogram of the evident normal brain (PDF 141 kb) [file 10334_2016_599_MOESM2_ESM.pdf]

**A**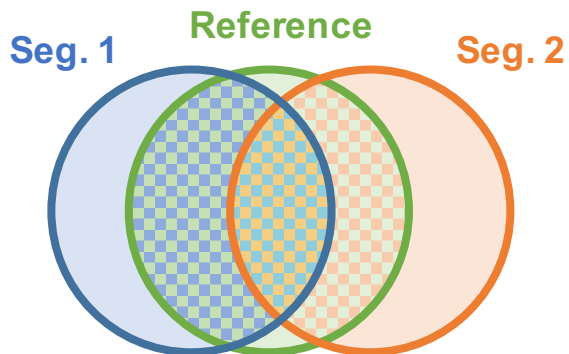**B**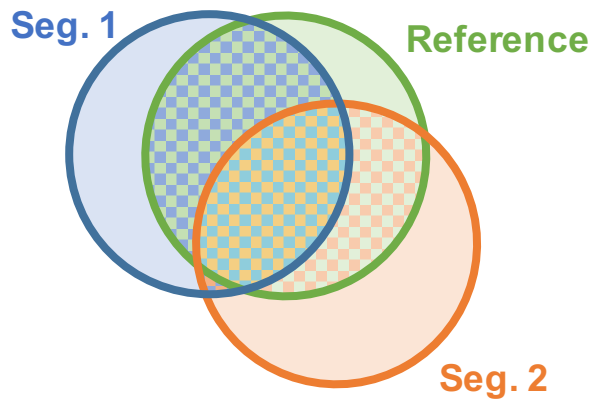**C**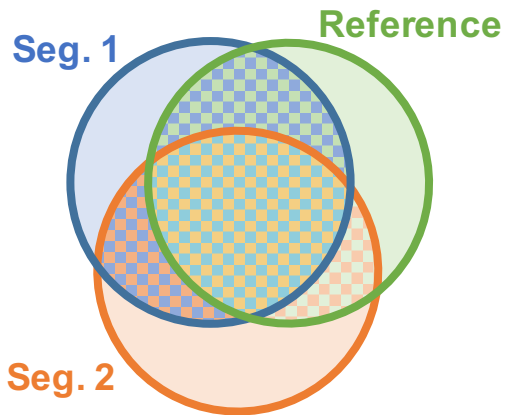**D**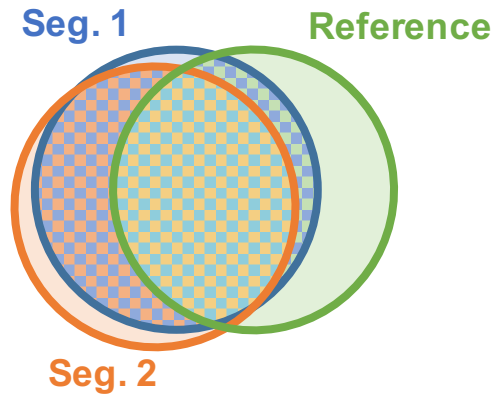

Supplement: Supplementary file 3 — Fig. S2 Similarity of two segmentations to a third reference segmentation does not guarantee thesimilarity between the first and the second segmentation. Segmentation 1 and 2 have equal Dicesimilarity index with reference segmentation, but they have A) low similarity; B) average similarity C)high similarity D) very high similarity (PDF 46 kb) [file 10334_2016_599_MOESM3_ESM.pdf]

Comparison between volumes ratios extracted from different combinations of sequences

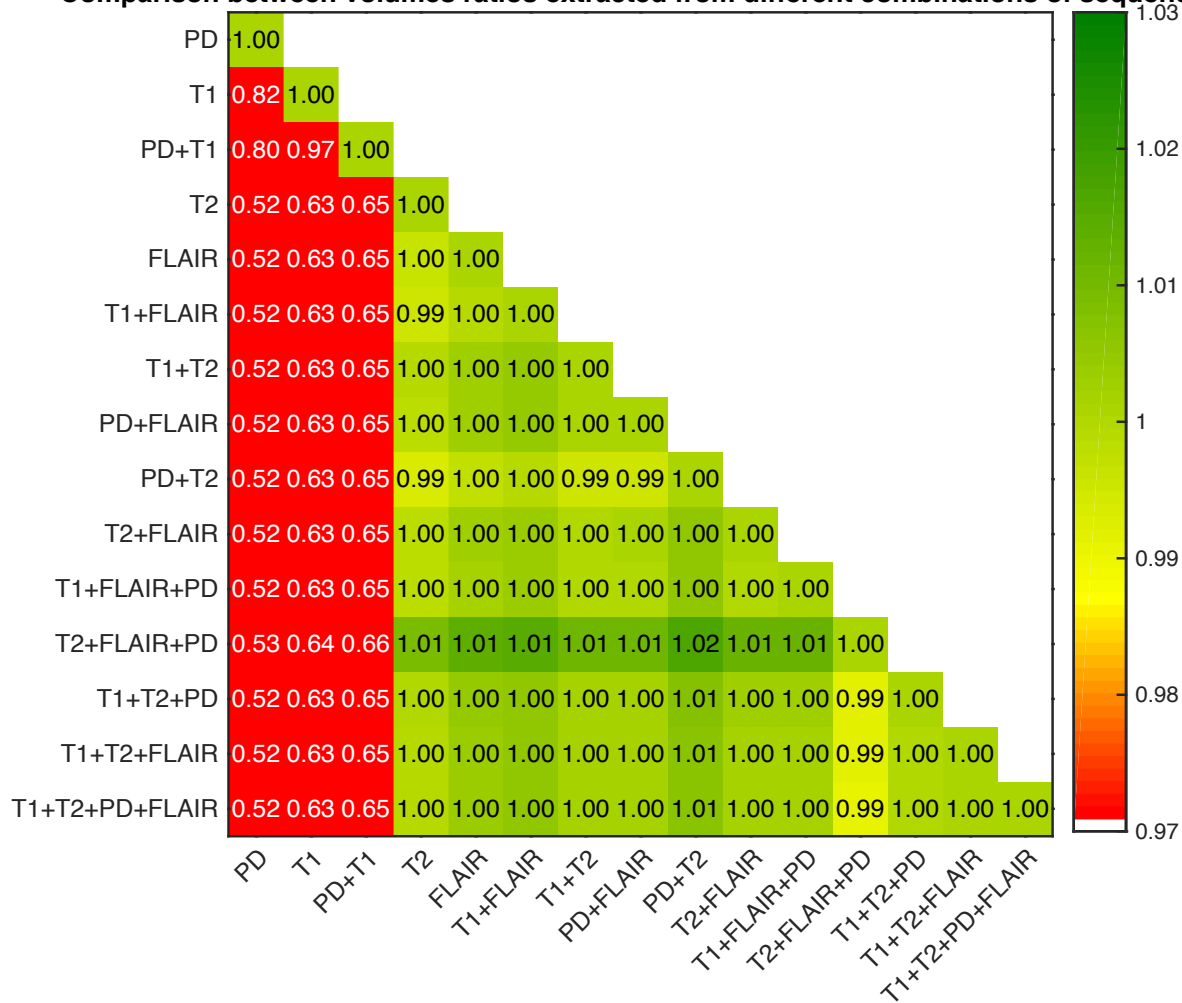

Supplement: Supplementary file 4 — Fig. S3 Ratio between white matter hyperintensities (WMH) volume when measured using differentinput sequences, comparing results from CASCADE using different input sequences to one another(Volume estimated from input combination indicated in the horizontal axis divided by the one in thevertical axis) (PDF 75 kb) [file 10334_2016_599_MOESM4_ESM.pdf]

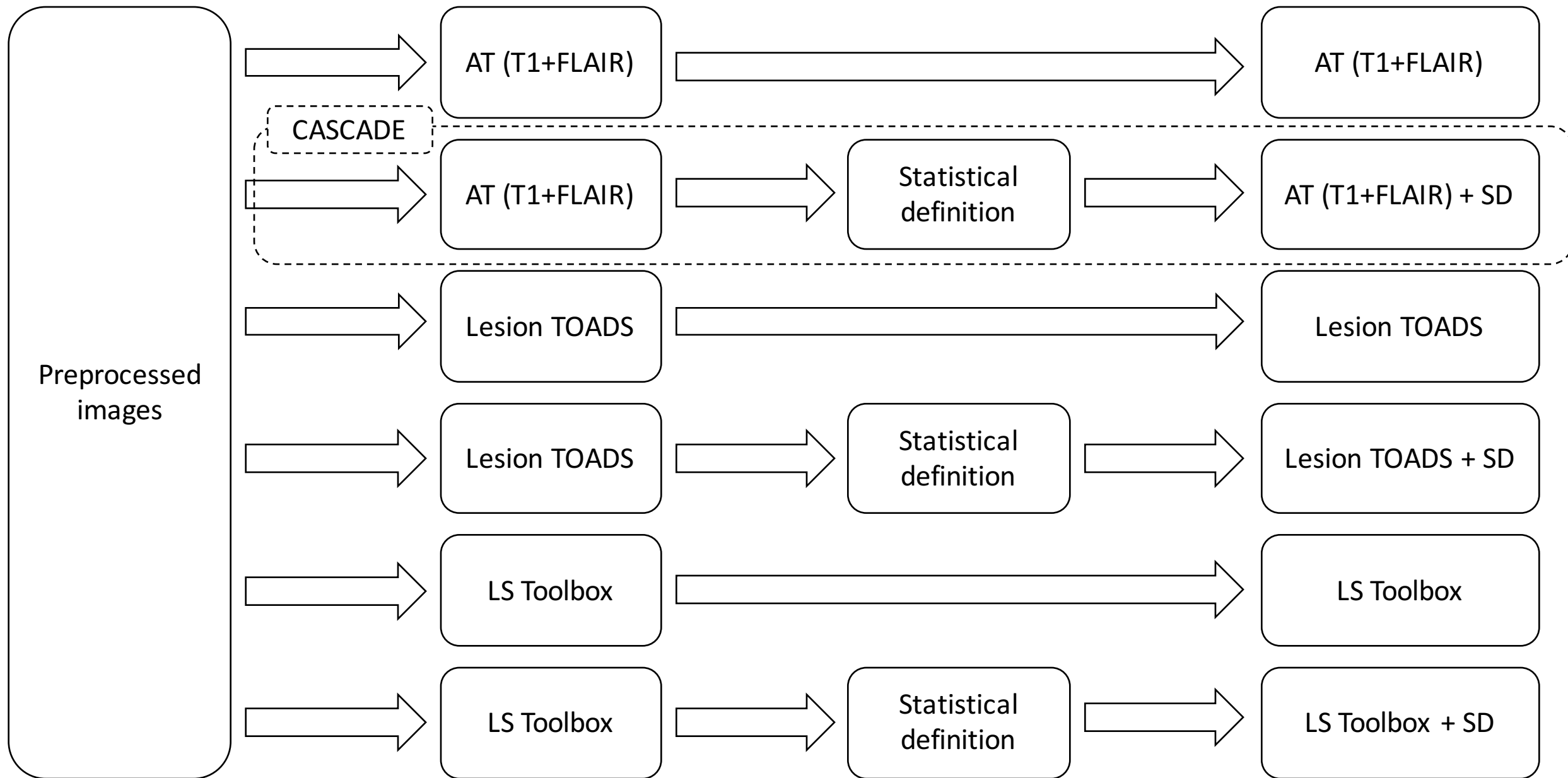

Supplement: Supplementary file 5 — Fig. S4 Processing design to analyze the effect of using different algorithms for detecting “evidentlynormal brain” All inputs use T1 and FLAIR as the input sequence. The dashed line is the procedureused in the CASCADE implementation, used to create the results in the paper. AT: Adaptive threshold,SD: Statistical definition (PDF 14 kb) [file 10334_2016_599_MOESM5_ESM.pdf]

**Dice coefficients using different methods for detecting evidently normal brain.**

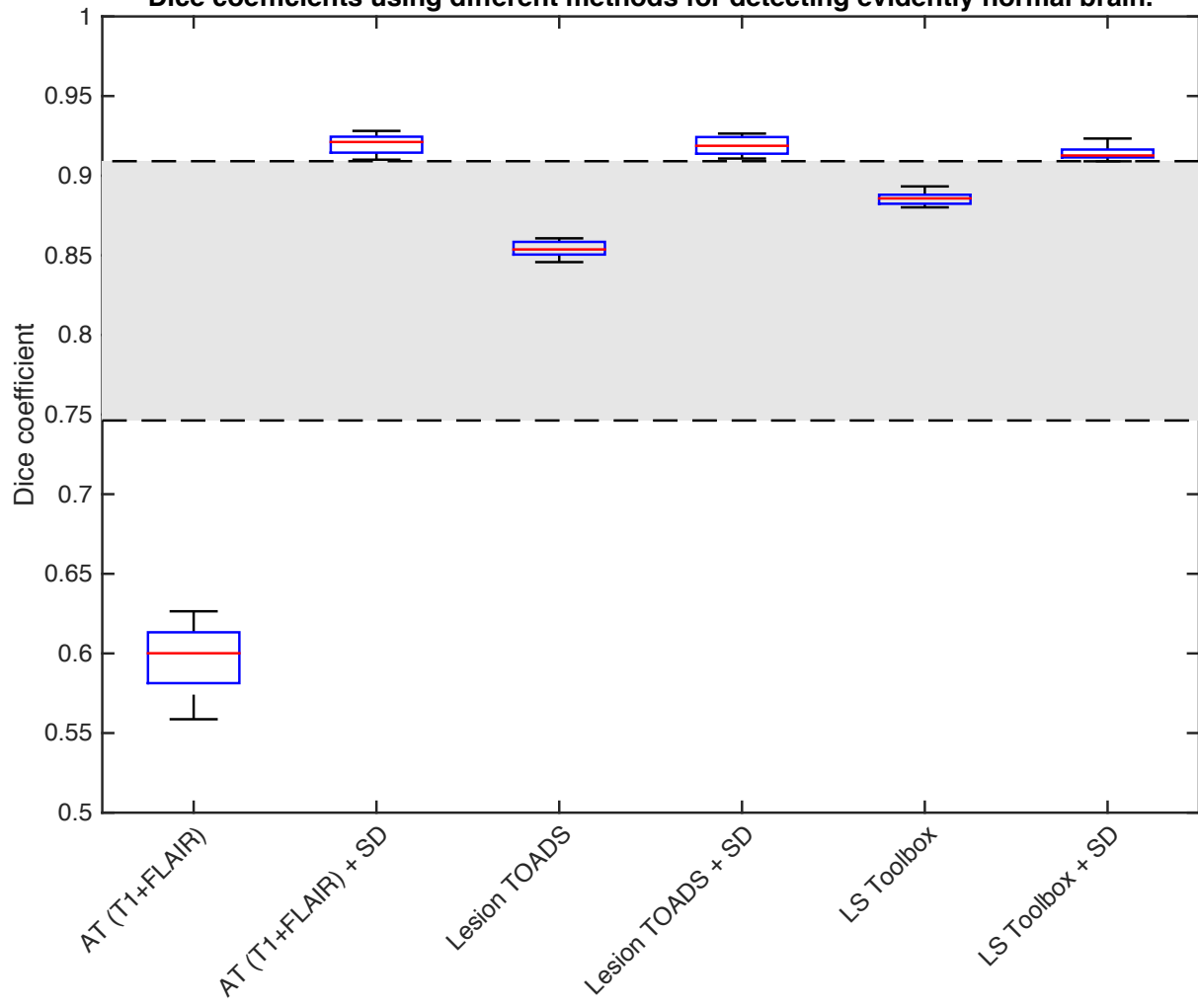

Supplement: Supplementary file 6 — Fig. S5 Comparison between the results of WMH segmentation using different methods for detectingevidently normal brain. All inputs use T1 and FLAIR as the input sequence. AT: Adaptive threshold,SD: Statistical definition (PDF 22 kb) [file 10334_2016_599_MOESM6_ESM.pdf]
