# Supplementary material for: Reproducible segmentation of white matter hyperintensities using a new statistical definition
Source: MAGMA. 2016 Dec 9;30(3):227–37. doi: 10.1007/s10334-016-0599-3 (PMC5440501; doi:10.1007/s10334-016-0599-3)
Supplement: Supplementary file 7 — Table S1 (PDF 56 kb) [file 10334_2016_599_MOESM7_ESM.pdf]

Table S1 Different components of the approaches to the segmentation of WMH

Approach	Input	Segmentation Agent	Segmentation Target	By product
Manual	MRI	Human	Compliance with guideline	–
Supervised machine learning	MRI + Human	Computer	Similarity to manual	Reproducibility
Unsupervised machine learning	MRI	Computer	Similarity to manual	Reproducibility
Statistical definition	MRI	Computer	Reproducibility	Similarity to manual
